# REMD Simulations of Full-Length Alpha-Synuclein Together with Ligands Reveal Binding Region and Effect on Amyloid Conversion

**DOI:** 10.3390/ijms231911545

**Published:** 2022-09-30

**Authors:** Pavel I. Semenyuk

**Affiliations:** Belozersky Research Institute of Physico-Chemical Biology, Lomonosov Moscow State University, Leninskie Gory 1/40, 119991 Moscow, Russia; psemenyuk@belozersky.msu.ru

**Keywords:** alpha-synuclein, aggregation inhibitor, molecular modeling, amyloid conversion, intrinsically disordered proteins, binding site prediction

## Abstract

Alpha-synuclein is a key protein involved in the development and progression of Parkinson’s disease and other synucleinopathies. The intrinsically disordered nature of alpha-synuclein hinders the computational screening of new drug candidates for the treatment of these neurodegenerative diseases. In the present work, replica exchange molecular dynamics simulations of the full-length alpha-synuclein together with low-molecular ligands were utilized to predict the binding site and effect on the amyloid-like conversion of the protein. This approach enabled an accurate prediction of the binding sites for three tested compounds (fasudil, phthalocyanine tetrasulfonate, and spermine), giving good agreement with data from experiments by other groups. Lots of information about the binding and protein conformational ensemble enabled the suggestion of a putative effect of the ligands on the amyloid-like conversion of alpha-synuclein and the mechanism of anti- and pro-amyloid activity of the tested compounds. Therefore, this approach looks promising for testing new drug candidates for binding with alpha-synuclein or other intrinsically disordered proteins and at the same time the estimation of the effect on protein behavior, including amyloid-like aggregation.

## 1. Introduction

Alpha-synuclein is a key protein involved in the development and progression of Parkinson’s disease and other synucleinopathies [1,2,3]. It is an intrinsically disordered protein, normally distributed in presynaptic terminals in an equilibrium between membrane-bound and soluble forms but prone to amyloid-like aggregation with the formation of toxic oligomers and fibrils [4,5]. The mechanism of this process is very complicated and believed to start with the amyloid conversion from a native form to a beta-structural one, followed by the formation of pre-fibrillar soluble oligomers and fibrils [6,7]. Alpha-synuclein comprises 140 amino acids and includes the so-called non-amyloid-beta component (NAC) domain which forms the main part of beta-structured fibrils and plays a key role in fibril formation [3,8,9]. Surprisingly, most known mutations associated with familial Parkinson’s disease are located outside of this domain [10]. The molecular mechanism of the effect of these mutations is unclear. The same is true for ligands with observed anti- or pro-amyloid activity such as fasudil [11], phthalocyanine tetrasulfonate [12], and polyamines [13]; all of these compounds were shown to bind outside of the NAC domain but somehow strongly alter fibril formation.

A range of various compounds was suggested as putative inhibitors of alpha-synuclein fibrillization but for most of them, the mechanism of action and even the binding site are unclear [14,15,16]. The disordered nature of alpha-synuclein hinders the search for new anti-amyloid drug candidates in silico. There are two types of data on their structure derived from NMR: a partially structured state formed in SDS micelles, which is usually considered a membrane-bound state [17], and an ensemble of conformations that appears in solution [18]. Few papers have described conventional docking to the membrane-bound structure [19,20]. However, the correlation between the binding of ligands with this form of alpha-synuclein and its putative effect on the formation of amyloid fibrils is questionable. An alternative approach is ensemble docking—a docking of a ligand to a set of prevalent conformations of a protein [21,22,23]. Unfortunately, though there are some examples of usage [24,25], this approach is not ideal for completely disordered proteins such as alpha-synuclein. First, if the ensemble of conformations is large and wide (more than 500 conformations in the case of alpha-synuclein), it requires complicated computations and analysis and, therefore, deals with a limited number of conformations. Second (and more important), a conventional docking to each of the conformations may be incorrect because of the big lability of the protein and therefore the dynamic nature of the binding. On the other hand, molecular dynamics simulations might be useful for studying the amyloid-like conversion and aggregation of proteins or their fragments [26,27,28,29,30,31].

Here, we report an atomistic replica exchange molecular dynamics simulation of full-length alpha-synuclein in the presence of small-molecule ligands. Three ligands differed in structure and nature with the reliably established binding site: fasudil, phthalocyanine tetrasulfonate, and spermine. The first two are known to inhibit alpha-synuclein fibrillization [11,32], whilst polyamines promote it [13,33]. Because of the importance of residues located outside the NAC domain, full-length alpha-synuclein was used. In addition to a successful prediction of the binding site, realistic insights into the mechanism of the ligand binding effect on amyloid conversion were achieved.

## 2. Results

### 2.1. Predicted Binding Sites Agree Well with Experiments

Three diverse compounds with a well-established anti- or pro-amyloid activity of the binding site, namely, fasudil, phthalocyanine tetrasulfonate (PcTS), and spermine, were tested for the binding to full-length alpha-synuclein using a replica exchange molecular dynamics simulation; the systems contained a single chain of alpha-synuclein and a molecule of one of the ligands. All three ligands efficiently interacted with the intrinsically disordered form of the protein.

**Figure 1:**
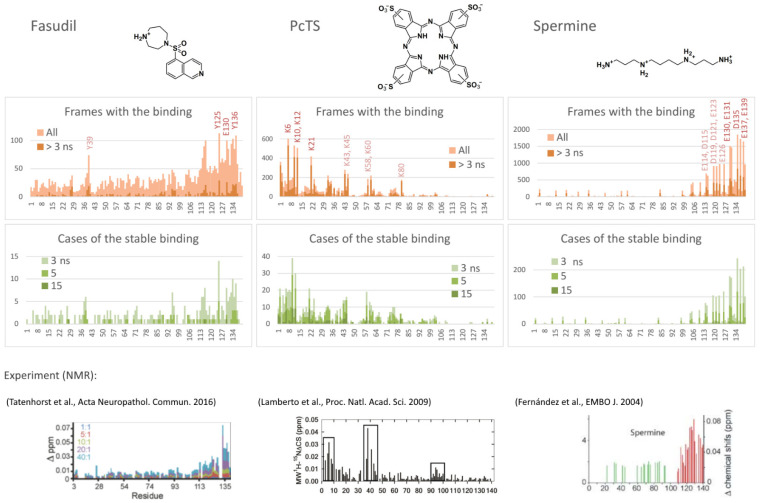
Binding profiles of fasudil (**left**), PcTS (**center**), and spermine (**right**); number of all or stable frames with binding to a particular residue, number of cases of continuous binding for at least 3, 5, or 15 ns, and experimental data of changes in the mean weighted chemical shifts reprinted with permission from [11,12,13].

The binding probability profiles were estimated as a number of frames with the ligand bound to a particular residue to enable the selection of the preferred binding residues and, consequently, to predict the binding site. As seen in Figure 1, the binding propensity varies substantially among the alpha-synuclein chain. Since almost all protein regions have residues with a non-zero score, all regions of the protein chain were exhaustively explored by the ligand, and therefore, the different occupations of the found potential binding sites enable the suggestion of an optimal one. For a better understanding of the binding nature and stability, the profiles were filtered to remove unstable binding cases (with a binding time less than 3 ns); in addition, profiles of the cases of continuous binding (with different thresholds for the binding time) instead of frames were calculated. Analysis of these profiles resulted in the selection of the most efficiently interacting residues.

Thus, **fasudil** preferred to interact with C-terminal residues Y125, Y130, Y133, Q134, and Y136 (Figure 1, left), which agrees well with experimental data from [11] (Figure 1, bottom). An additional putative binding site, Y39, which is not important for the binding according to [11] but demonstrated some interaction in another experiment [34], showed a relatively high number of total binding frames in the model, but most of the binding cases were unstable, suggesting that the binding affinity is smaller compared to the main binding site (residues 125, 130, and 133–136).

**Figure 2:**
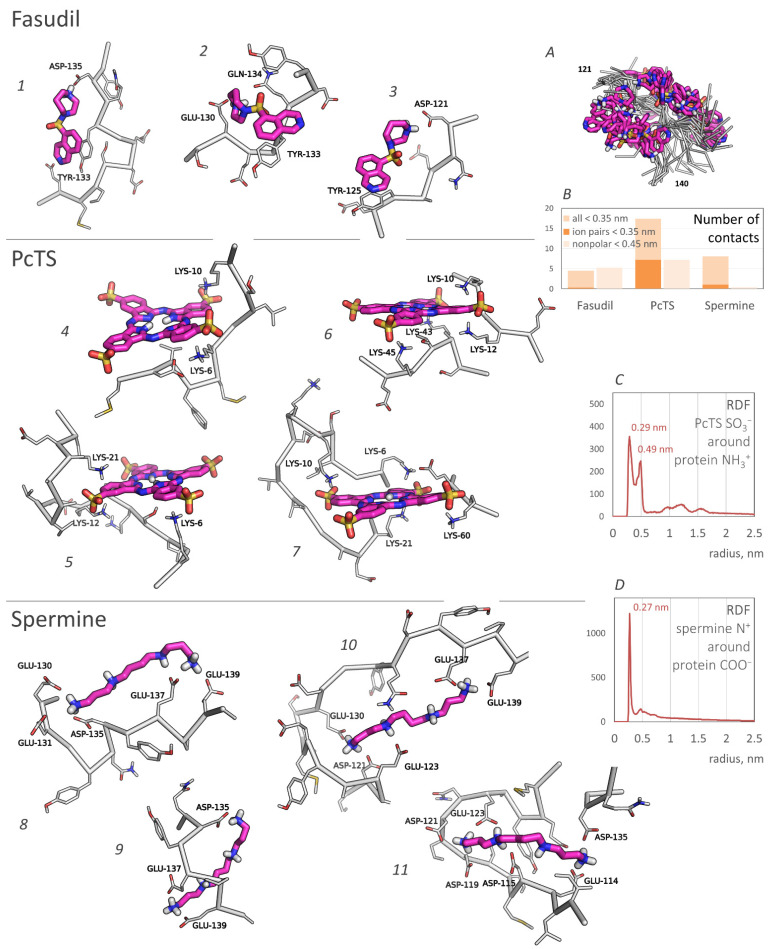
Typical binding poses of fasudil, PcTS, and spermine. The ligands are shown in purple; nonpolar hydrogens are hidden. The poses (1–11) are numbered for the convenience of mentioning. Inset (**A**) shows the superimposed binding poses of fasudil bound to the alpha-synuclein 133–136 region (only a backbone of the protein chain is shown); (**B**) represents a number of contacts (pairs) between the alpha-synuclein chain and the ligands; (**C**,**D**) represent the RDF profiles of PcTS SO_3_^−^ groups (specifically, oxygen atoms) around the positively charged amino groups of alpha-synuclein (**B**) and the positively charged amino groups of spermine around the carboxyl groups of alpha-synuclein.

According to the binding profiles, the interaction of fasudil and alpha-synuclein is very dynamic. Many contacts of even top-rated residues were relatively unstable. Furthermore, lots of different positions of the fasudil molecule were observed. Generally, fasudil formed a stacking interaction with tyrosine residues (Y125 or Y133) and electrostatic interaction with an acidic residue (E130, D135, or another one) (Figure 2, poses 1–3). Notably, even when bound to the 133–136 region of alpha-synuclein, the fasudil molecule does not occupy a stable position but dynamically moves around this part of the binding site (Figure 2A). Thus, the binding was stable but dynamic since fasudil formed new contacts with alpha-synuclein after breaking previous contacts.

**PcTS**, being a polyanion, interacted with positively charged groups of alpha-synuclein, namely, the amino groups of lysine residues. N-terminal residues K6, K10, K12, and K21 served as top-rated binding residues; K32, K34, K43, K45, K58, K60, and K80 can also participate in the binding but less efficiently (Figure 2, center). Generally, PcTS formed bonds with 2–3 lysine residues from the main binding site (residues 6, 10, 12, and 21) (Figure 2, poses 4–5) and sometimes 1–2 additional bonds with other lysine residues, including those located in the NAC-domain (Figure 2, poses 6–7). Figure 2B (prevalence of ion pairs) and Figure 2C (the radial distribution function of sulfonate groups of PcTS around amino groups of alpha-synuclein) corroborate the electrostatic nature of the binding. All main contacts between PcTS and alpha-synuclein residues were stable in contrast to those of fasudil. A stacking interaction with aromatic residues (mainly F4 and Y39) was rare and does not seem to play a significant role in the binding. The predicted binding region agrees well with the experimental data (Figure 1, bottom, and [12]) but the particular binding site does not. Lamberto et al. demonstrated a key role of F4 and Y39 for the binding, although the binding caused changes in the mean weighted chemical shifts for a broad region at the N-terminus of alpha-synuclein; furthermore, they showed the importance of an electrostatic interaction with lysine residues. Thus, the F4A/Y39A mutant still bound PcTS via the N-terminal region. In summary, one can conclude that our model correctly predicted the binding region, including a strong prevalence of N-terminal lysine residues compared to other charged regions, but only a part of the preferred binding residues, though key residues, F4 and Y39, were not recognized.

Finally, **spermine** interacted with the C-terminal region of alpha-synuclein enriched with acidic residues. The binding was very efficient and occurred in almost 94% of frames, in contrast to approximately a quarter in the case of fasudil and PcTS. The alpha-synuclein residues E130, E131, D135, E137, and E139 were the most preferred (Figure 1, right); the residues E114, D115, D119, D121, E123, and E126 also can participate in the binding. This result agrees with experimental data from [13] which assigned the binding site to C-terminal residues 109–140 and indicated changes in chemical shifts for numerous residues in this region (Figure 1, bottom), although the residues A124, M127, and S129 considered by Fernandez et al. as important for the binding did not show a significant binding propensity in our model. The binding was electrostatically driven (Figure 2B,D): a polycationic molecule of spermine simultaneously interacted with 3–4 carboxyl groups of alpha-synuclein, giving a preference to the main binding site, namely, residues 130–131, 135, 137, and 139 (Figure 2, poses 8–11).

In summary, one can conclude that the binding region was correctly predicted for all three ligands with an accurate assignment of the binding residues for fasudil and spermine but only partially for PcTS.

### 2.2. The Ligand Binding Modulates Conformational Ensemble and Amyloid Conversion of Alpha-Synuclein

**Figure 3:**
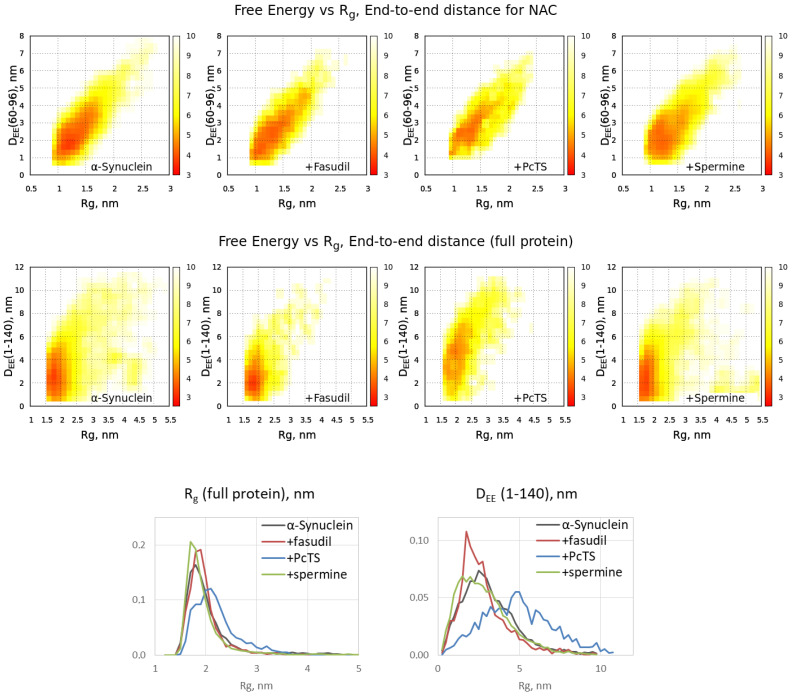
Free energy distributions for conformational ensembles of alpha-synuclein in a free form and when bound with ligands: NAC domain (**top**) and full protein chain (**middle**) as well as Rg and end-to-end distance distributions for the ensembles (**bottom**).

The binding of the ligands might alter the behavior of the full-length alpha-synuclein chain. According to the free energy surfaces of alpha-synuclein in a space {Radius of gyration (R_g_), distance between terminal C-alpha atoms}, the changes caused by binding with fasudil and spermine are not pronounced, whereas the binding with PcTS significantly shifts the peak on the distribution to a more expanded conformation (Figure 3). This effect is the most pronounced for the full-length alpha-synuclein chain, whilst the conformational ensemble of the NAC domain changes less. Distribution of the conformations on R_g_ and end-to-end distance is significantly shifted to higher values in the case of PcTS compared to the free form of alpha-synuclein as well as that bound to fasudil and spermine (Figure 3, bottom), indicating further expansion of the alpha-synuclein chain.

**Figure 4:**
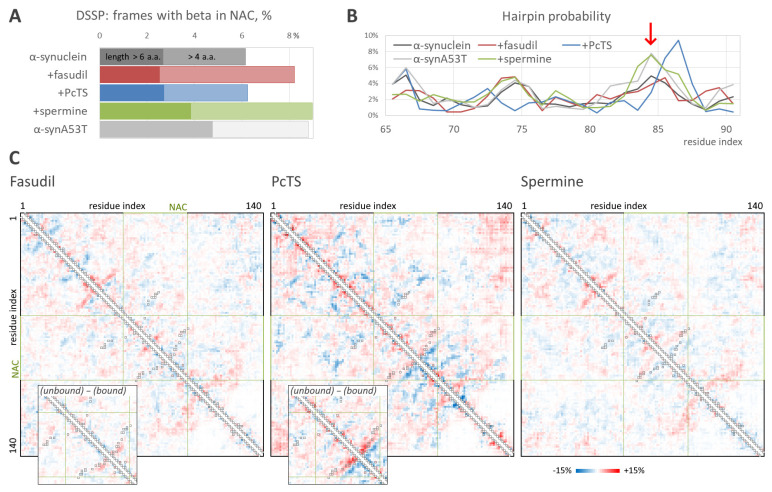
Propensity to form beta-hairpin. (**A**). Percentage of conformations with prolonged (dark and light colors shows values for a length threshold of 6 or 4 residues, respectively) beta-elements, according to DSSP, in the absence and the presence of the ligands as well as in the free mutant form, A53T. (**B**). Hairpin formation probability versus residue index in NAC-domain according to the distance from a reference beta-hairpin; red arrow indicates the hairpin position specific for fibrils. (**C**). Differential intramolecular contact maps obtained as the difference between contact maps for free alpha-synuclein ensemble and alpha-synuclein bound with the ligands; red and blue indicate contacts prevalent in the free form and the bound form, respectively. Insets show differences between the contact map fragments of alpha-synuclein unbound and bound with the ligand in an overall simulation ensemble for fasudil and PcTS. Black squares mark the contacts presented in the structure of alpha-synuclein fibrils, PDB ID 2n0a.

The structural ensembles obtained in the REMD simulations enable the estimation of residue-residue intramolecular interactions and the propensity to form beta structures such as beta-hairpins. According to DSSP analysis, the binding of spermine caused the enhanced formation of beta-structured elements in the NAC domain (Figure 4A) with an efficiency very similar to that in the case of free aggregation-prone alpha-synuclein mutant A53T; the formation of relatively short beta-structures (but not of longer ones) was also observed for fasudil. However, the beta-hairpin formation probability, estimated by the root mean square distance from a reference 12-residue beta-hairpin, was significantly higher in the system with the bound spermine compared to the free alpha-synuclein. The hairpins were formed by the region around residues 83–86 (Figure 4B, red arrow), which is a key part of the beta-sheet in alpha-synuclein amyloid fibrils, indicating a pro-amyloid action of spermine. Furthermore, the hairpin probability profile in the presence of spermine almost coincided with that for the A53T mutant. Surprisingly, PcTS binding resulted in an increased hairpin formation propensity by the region around residues 86–87, although the overall hairpin formation was smaller. This data correlates with a significant alteration of the conformational ensemble of alpha-synuclein induced by PcTS binding (Figure 3) and with a significant change in the residue-residue contacts. Indeed, hairpins around residues 83–85 (recognized on a contact map as a near-diagonal line perpendicular to the map diagonal) are less preferable (the difference is up to 7%) in the ensemble with the bound PcTS compared to the reference ensemble of free alpha-synuclein (Figure 4C), whereas hairpins around residues 86–87 are significantly more probable with a difference up to 11.5%. The decreased propensity to form hairpins around residues 83–85 is more noticeable when comparing alpha-synuclein conformations bound and unbound with PcTS in the system with the ligand.

Changes in alpha-synuclein residue-residue intramolecular contacts induced by the binding of fasudil and spermine are much less pronounced. However, contacts corresponding to the fibril-like beta-hairpin around residues 83–85 are 3–5% more frequent in the form bound with spermine. Since the conformational ensemble of alpha-synuclein was broad, and only a part of conformations can be considered beta-like (Figure 4A), the difference does not look insignificant. This tendency agrees with an elevated propensity of alpha-synuclein to form fibrils in the presence of spermine.

In summary, the analysis of the conformational ensembles suggests a putative effect on alpha-synuclein behavior. In the case of fasudil, an unambiguous conclusion cannot be made, although the fact that beta-structure-associated features, such as the hairpin formation propensity profile and intramolecular contacts, are similar to those for free alpha-synuclein suggests non-pro-amyloid action. PcTS binding significantly alters the conformational ensemble of alpha-synuclein, although the type of effect cannot be predicted from this model. On the contrary, the elevated propensity to form beta-structures, including beta-hairpins in the region, which forms a beta-sheet in real fibrils, indicates a pro-amyloid action of spermine.

## 3. Discussion

In summary, the suggested approach enables the accurate prediction of the binding site for low-molecular ligands on full-length alpha-synuclein. A replica exchange molecular dynamics simulation of the system containing full-length intrinsically disordered alpha-synuclein together with a ligand molecule gave a realistic conformational ensemble with plenty of conformations bound to the ligand. Despite the variety of the binding poses, statistical analysis enables the recognition of the binding site, which coincided with the binding site obtained using experiments in the case of fasudil and low-molecular polyamine. As to PcTS, the prediction of particular binding residues was not very accurate, though the binding region was predicted correctly.

Simulation results enable the estimation of binding affinity in addition to binding site prediction. Among the three tested ligands, spermine was shown to exhibit the highest affinity; an overall tendency can be summarized as Fas < PcTS < Spr based on the number of all bound cases. This regularity does not clearly fit the experimental data: K_d_ values of 3.1 × 10^−6^ M and 6.2 × 10^−4^ M for PcTS [32] and spermine [13], respectively. As for fasudil, the estimation from concentration dependence of the chemical shift in [11] gives a value in a millimolar range similar to a simulated K_d_ value (3.0 × 10^−3^ M [34]). These values represent the regularity Fas < Spr < PcTS. In other words, our model underestimated the binding affinity of PcTS. Regardless of the possible difference in experimental conditions in the mentioned studies, it probably arises from the unideal prediction of the binding: the binding region was properly predicted but the particular binding residues were only partially recognized (the prediction included a correct recognition of lysine residues but not F4 and Y39). This might be due to the unideal parametrization of the PcTS molecule, which is very peculiar for biology-oriented force fields. On the other hand, PcTS binding cases were less numerous but much more stable and formed more contacts between the protein and the ligand compared to those of spermine and fasudil, suggesting a higher binding affinity.

The obtained model provided molecular insight valuable for understanding the mechanism of the anti-amyloid action of fasudil and PcTS as well as the pro-amyloid action of spermine, although it is not enough for a clear understanding of these mechanisms. It is of special interest how the binding could influence fibril formation, which strongly depends on the NAC domain, if all three tested compounds were shown to bind out of the NAC domain. Fasudil has been shown to interact with the C-terminal region of alpha-synuclein, affecting the regions responsible for binding metal ions, and is thus linked with amyloid conversion [35]. The importance of Y136, which is a key part of the binding site of fasudil, for alpha-synuclein amyloid conversion is indirectly indicated by the fact that the Y136C mutant forms amyloid fibrils inefficiently [36]. A probable link between post-translational modifications of other fasudil-binding residues, Y125, Y133, and Y136, and Parkinson’s disease progression [10] also suggests that the interfering behavior of these residues by fasudil might alter the aggregation process. However, the obtained conformational ensemble of the alpha-synuclein bound to fasudil does not differ much from that of free alpha-synuclein, and the propensity to form beta-hairpins or other beta-structures is similar to that of free alpha-synuclein. On the contrary, the binding of PcTS to the N-terminal positively charged residues strongly altered the structural ensemble of alpha-synuclein, inducing expansion of the protein chain. Since the formation of intramolecular contacts and loops is an important step of amyloid conversion, expanding the chain should inhibit amyloid conversion and therefore inhibit fibrillization. The formation of amyloid-like hairpins in the NAC region was also altered due to PcTS binding. Finally, the binding of spermine significantly enhanced the propensity of alpha-synuclein to form beta-hairpins in NAC regions, indicating the pro-amyloid action of the spermine. It is also corroborated by the coincidence of the values corresponding to beta-hairpin probability and its most probable positions in the ensemble obtained in the presence of spermine and the ensemble of free fibrillation-prone alpha-synuclein mutant A53T. The elevated propensity to beta-hairpin formation due to spermine binding seems to arise from the binding with multiple C-terminal acidic residues. Of note, the binding region was relatively prolonged, and the one spermine molecule often interacted simultaneously with residues located relatively far from each other (Figure 2, poses 10–11). The formation of loops caused by spermine binding is also indicated by a slightly decreased end-to-end distance of alpha-synuclein (Figure 3, bottom). Furthermore, one can hypothesize that a single spermine molecule, being a short polycation, could interact with the mentioned acidic residues in two molecules of alpha-synuclein. Therefore, the action might be similar to the action of polyvalent metal cations, which are known to accelerate amyloid conversion and aggregation by promoting intra- and intermolecular contacts [29,37]. This hypothesis is corroborated by overlapping the binding site of spermine and the metal ions-binding region, namely, residues 119–125 [35,38]. In addition, spermine binding might affect electrostatic interactions, which are known to be important for amyloid aggregation [38,39].

The binding of all three tested ligands is highly dynamic. The ligand molecule forms many contacts with the protein but most of them are relatively unstable and often switch to each other. In the other words, the ligand molecule is stably bound but the exact pose is dynamically changing. Such a dynamic movement of the ligand molecule among preferably binding residues was especially conspicuous in the case of fasudil and agrees well with a “dynamic shuttling” observed earlier in conventional simulations of alpha-synuclein and fasudil or similar molecules [34]. The binding of spermine and especially PcTS were significantly more stable (Figure 1), but different binding cases gave different sets of the bound residues, indicating multiple local minima on energy profiles. Regardless, taking into account this behavior of all three ligands, one might consider the term “binding site” incorrect since the residues of this site do not bind the ligand together and permanently: only a part of them forms contacts with the ligand simultaneously, and the unbound residues of the binding sites might be located far from the ligand. From this point of view, it would be more accurate to specify the preferred binding residues instead of the binding site and mention that the ligand molecule can move among these residues or even sub-sites.

A very dynamic pattern of the alpha-synuclein binding with ligands might reflect a common nature of alpha-synuclein. The present study demonstrated a dynamic binding (in terms of contact lifespan) of fasudil and a kind of dynamic binding (more stable but with various contacts) of PcTS and spermine. We suggest this regularity can take place for many other ligands as well. Indeed, the conformational ensemble of free alpha-synuclein obtained in our REMD simulation shared a broad distribution of conformations with a very limited capacity for clustering. The same is true for the initial ensemble retrieved from the protein ensemble database. The binding with the ligands did not narrow the distribution, suggesting a broad nature of free energy landscapes without any sharp minimums. Thus, alpha-synuclein can be considered as a “truly disordered” protein, and the binding of the tested ligands seems to not stabilize some conformations or somehow order them.

From this point of view, the anti-amyloid action of tested ligands is not a result of the stabilization of alpha-synuclein in a single conformation. Instead, the ligands seem to alter the conformational ensemble and therefore shift the equilibrium to a monomeric form. Such action looks promising for biological systems because of a likely lower effect on alpha-synuclein interaction with other macromolecules compared to the stabilization of a particular conformation.

When discussing the probable limitations of the approach for other intrinsically disordered proteins or other ligands, one should mention a system size, i.e., the length of a protein. Prediction of the ligand-binding site and understanding of the binding mechanism is even more challenging to the realism of the obtained conformational ensemble than the modeling of the free protein. As a result, it requires very powerful computing resources. We demonstrated a high potency of this approach even for 140-a.a. protein, alpha-synuclein, which is completely disordered, and expect that it should give a great result for shorter proteins or peptides. However, despite obtaining valuable information about the amyloid-like conversion in the presence of the ligands, a clear understanding of the influence of binding on the behavior of the complex seems to require much longer simulations and is computationally consuming. In addition, only the initial states of amyloid conversion can be simulated since modeling even two alpha-synuclein molecules (which definitely is not enough to model aggregation) increases the computational cost exceptionally. On the contrary, it might be promising for smaller proteins such as amyloid beta peptides and can give more data about the ligand binding and effect of the binding on protein behavior compared to a long conventional molecular dynamic simulation.

## 4. Methods

### 4.1. System Construction and Simulation

**Figure 5:**
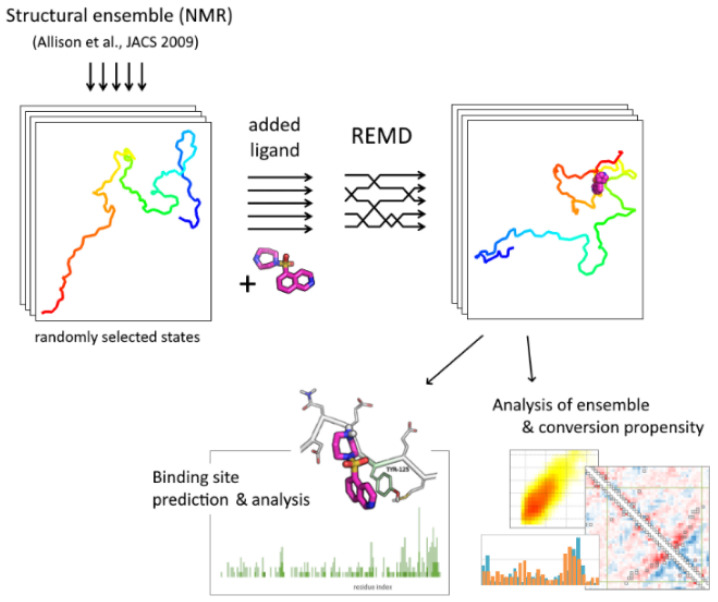
The scheme of the REMD-based docking pipeline.

An overall pipeline of the approach is presented in Figure 5. Initial structures for replica exchange molecular dynamic simulations (84 conformations) were randomly selected from the structural ensemble [18] retrieved from the Protein Ensemble Database (proteinensemble.org). A single molecule of alpha-synuclein in a protonation state corresponding to a physiological pH value was added to the system in different conformations. After configuring simulation boxes of the same size, a single ligand molecule was added to all conformations in a random position. Then, the system was solvated with an equal number of water molecules and neutralized by counter-ions. The sampling included 84 replicas with a temperature range of 300–360 K; temperature values were determined using a temperature generator [40]. After energy minimization and a short equilibration at particular temperatures, main simulations were performed for 100 ns with a step of 2 fs; replica-exchange attempts were performed every 500 steps. The replica exchange probability was 0.22–0.28.

REMD simulations were performed in GROMACS 2018.8 software [41] using CHARMM36m force field [42]; molecules of ligands were parametrized using CGenFF service [43]. Periodic boundary conditions and the particle mesh Ewald method for handling long-range electrostatic interaction were used. NPT ensemble, V-rescale thermostat, and a Parrinello–Rahman barostat were used.

A reference simulation with free alpha-synuclein was performed in a similar manner but without the addition of the ligands. In addition, a simulation with a free A53T mutant was performed.

### 4.2. Binding Site Prediction

The frequency of contacts between the ligand and particular residue of alpha-synuclein was determined from a gross number of frames where the corresponding distance between heavy atoms was less than 0.35 nm; in addition, the frames (or cases of the binding) were filtered to exclude non-stable contacts with a threshold of 3–15 ns. The number of different replicas where the particular contact is stable was also determined. Radial distribution functions (RDF) were calculated for oxygen atoms of PcTS SO_3_^−^ groups around the positively charged amino groups of lysine in alpha-synuclein and for the positively charged nitrogen atoms of spermine around the oxygen atoms of the carboxyl groups in alpha-synuclein.

### 4.3. Conformational Ensemble Analysis

Free energy distributions for structural ensembles were determined as –ln(P_x,y_) for every segment {x, y} after slicing overall intervals.

The occurrence of beta-structured elements was estimated in two ways. First, the probability of beta-element formation in the NAC region according to DSSP was compared. Second, the root mean of the square distance between every frame and a reference beta-hairpin (PDB ID 2l8x, 12 a.a.) was calculated as a function of the beta-hairpin position in the NAC region. The percentage of frames with this distance lower than the threshold was used as a probability of hairpin formation at a particular position.

Protein-protein intramolecular contact maps were calculated for all frames with a time interval of 1 ns in all replicas; residues with a distance of between 0.15 nm and 0.5 nm between heavy atoms were considered as contacted ones. The difference between normalized contact maps for the ligand-bound conformational ensemble and the reference conformational ensemble (for free alpha-synuclein) is shown.

## 5. Conclusions

The replica-exchanged molecular dynamics simulation of an intrinsically disordered full-length alpha-synuclein together with low-molecular ligands in explicit water and counterions enabled an accurate prediction of the binding sites. Among the three tested compounds, binding site recognition showed a high accuracy for fasudil and spermine, and a moderate accuracy (with proper recognition of the binding region) for phthalocyanine tetrasulfonate, based on experimentally determined data. Analysis of the obtained structural ensembles for the alpha-synuclein/ligand complexes provided plenty of information about the protein behavior and a putative effect of the ligands on the amyloid-like conversion of alpha-synuclein. Thus, the anti-amyloid activity of phthalocyanine tetrasulfonate seems to be associated with a further expansion of the alpha-synuclein chain, as indicated by an increase in radius of gyration and end-to-end distance of the protein and a significant alteration of residue-residue intramolecular contacts. On the contrary, the binding of a pro-amyloid ligand, spermine, to the C-terminal acidic region of the alpha-synuclein accelerated the formation of beta-hairpins and other beta-structures, probably by promoting intra-molecular loops. Surprisingly, the binding of all three ligands, especially fasudil, had a dynamic nature and a limited site specificity: the ligand molecule can bound with a set of residues and exhibits a relatively flexible position in the binding site. Furthermore, the structural ensemble of the alpha-synuclein chain showed a broad distribution in the bound state as well as in the free state. Consequently, the activity of the ligands arises from the induced equilibrium shift instead of stabilization in any single (either low-amyloidogenic or highly amyloidogenic) conformation.

## Data Availability

Data on simulations are available upon request.
